# Sumoylation at the Host-Pathogen Interface

**DOI:** 10.3390/biom2020203

**Published:** 2012-04-05

**Authors:** Van G. Wilson

**Affiliations:** Department of Microbial & Molecular Pathogenesis, College of Medicine, Texas A & M Health Science Center, 8447 HWY 47, Bryan, TX 77807-1359, USA; Email: wilson@medicine.tamhsc.edu; Tel.: +1-979-436-0310; Fax: +1-979-436-0086

**Keywords:** virus, bacteria, SUMO, SIMs, immunity

## Abstract

Many viral proteins have been shown to be sumoylated with corresponding regulatory effects on their protein function, indicating that this host cell modification process is widely exploited by viral pathogens to control viral activity. In addition to using sumoylation to regulate their own proteins, several viral pathogens have been shown to modulate overall host sumoylation levels. Given the large number of cellular targets for SUMO addition and the breadth of critical cellular processes that are regulated via sumoylation, viral modulation of overall sumoylation presumably alters the cellular environment to ensure that it is favorable for viral reproduction and/or persistence. Like some viruses, certain bacterial plant pathogens also target the sumoylation system, usually decreasing sumoylation to disrupt host anti-pathogen responses. The recent demonstration that *Listeria monocytogenes* also disrupts host sumoylation, and that this is required for efficient infection, extends the plant pathogen observations to a human pathogen and suggests that pathogen modulation of host sumoylation may be more widespread than previously appreciated. This review will focus on recent aspects of how pathogens modulate the host sumoylation system and how this benefits the pathogen.

## 1. Introduction–Pathogens and Host Systems

Pathogens, especially intracellular ones like viruses, have evolved numerous mechanisms to counteract host defenses and to reshape the cellular environment into a permissive milieu that allows pathogen persistence and/or replication. Because of their limited genetic repertoires, viruses are highly efficient and typically encode proteins with multiple functions and which target critical cellular regulatory pathways to evoke maximal effects on the host cell. Well studied examples of viral manipulation of host systems include viral transcription factors that also regulate cellular gene expression [1], viral proteins that inactivate p53 to circumvent cell cycle arrest and apoptosis (reviewed in [2]), and viral proteins that drive cells into the proliferative phase (reviewed in [3]). An additional established theme is viral interaction with the host ubiquitin-proteasome system to regulate cellular activities through targeted degradation or protection of host proteins (reviewed in [4]). In many cases viral proteins co-opt cellular ubiquitin E3 ligases to re-direct substrate specificity or alter ligase activity. Alternatively, some viral proteins have intrinsic ubiquitin E3 ligase activity that directly promotes ubiquitination of selected host substrates. Both mechanisms lead to increased degradation of specific host proteins and thus diminish their available functional activity. In contrast, several well documented examples exist of viral proteins that have deubiquitinating activity, though in most cases their authentic substrates have not yet been identified. Presumably each of these viral deubiquitinating proteins removes ubiquitin from one or more host proteins thus decreasing their degradation and protecting host activities critical to viral pathogenesis or reproduction. In addition to the well-studied ubiquitin system, the ubiquitin super family includes a number of ubiquitin-like proteins including Atg8 [5], Nedd8 [6], ISG15 [7], and SUMO [8,9,10,11] that are all enzymatically conjugated to substrate proteins. Among these ubiquitin-like proteins, SUMO has been shown to modify numerous DNA and RNA virus proteins and is clearly an important host system during viral infection [12,13,14].

## 2. The SUMO System

Over the last 15 years, a new post-translational modification system has been defined that is enzymatically analogous to, but functionally distinct from the classical ubiquitin system [15,16]. This related system involves target protein modification of one or more lysine residues by conjugation of an ubiquitin-like, small polypeptide known as SUMO [17]. The human SUMO-1 gene encodes a 101 amino acid polypeptide with ~50% relatedness to the S. cerevisiae SMT3 protein and ~18% sequence relatedness to ubiquitin [8,10,11]. Vertebrate species have at least 2 additional genes for SUMO1 related proteins, SMT3A (SUMO2) and SMT3B (SUMO3) [18]. The three SUMO genes appear to form two subfamilies, as SUMO2 and SUMO3 share 87% sequence identity compared to only ~50% identity between SUMO2/3 and SUMO1. Less well studied is a fourth SUMO gene whose expression is restricted primarily to the kidneys, dendritic cells, and macrophages [19]; SUMO4 may play a role in diabetes [20], but overall its biological functions and activity are not well defined.

Like ubiquitination, sumoylation involves formation of a stable isopeptide bond between the ε-amino group of the target lysine and the carboxyl group of the C-terminal glycine of SUMO. As for ubiquitin, SUMO must first be proteolytically processed at its C-terminus, enzymatically activated, and then covalently attached via a thioester linkage to a cysteine residue of its conjugating enzyme, Ubc9. However, the SUMO proteases, the heterodimeric activating enzyme (Aos1/Uba2, called SAE1/SAE2 in this review), and Ubc9 are all specific for SUMO usage and do not function with ubiquitin [21,22]. Furthermore, transfer of SUMO to target proteins can occur directly from Ubc9, apparently without an absolute requirement for substrate-specific E3-type ligase enzymes that are necessary for ubiquitin addition [23]. As would be expected from this lack of an absolute E3 requirement, numerous target proteins for SUMO1 modification have been identified by their direct interaction with Ubc9 [8,9,21,24,25,26]. However, more recently it has become clear that there are SUMO-specific E3 ligases, and several types have been identified, including members of the PIAS protein family (Protein Inhibitors of Activated Stat) [27,28,29]; RanBP2, a nuclear pore protein [30]; the polycomb protein, Pc2 [31]; TOPORS, topoisomerase I binding protein [32], and members of the TRIM family [33]. Additional SUMO ligases are likely to be discovered and they undoubtedly will have important roles in regulating the functional biology of SUMO targets.

Lastly, the sumoylation state of target proteins is not static, but instead reflects a dynamic equilibrium between the forward process of SUMO addition and its removal by cellular desumoylating enzymes known as SENPs [34]. The mammalian SUMO proteases differ greatly in their sequences and are related primarily in the conserved region critical for cysteine protease catalytic activity. Furthermore, individual proteases have been shown to differ in intracellular localization with both nuclear [35,36] and cytoplasmic [37,38,39] species observed. The existence of multiple mammalian desumoylating enzymes, along with the demonstrated differences in intracellular distribution, suggests that desumoylation is likely to be a complex process that contributes to the regulation of activity of SUMO substrates.

Functionally, sumoylation is now implicated in a diverse array of critical cellular processes including nuclear processes such as RNA processing, chromatin remodeling, genome maintenance, transcriptional regulation, mitosis, meiosis, differentiation and development, apoptosis, and nucleocytoplasmic transport [40]. More recently, significant non-nuclear functions of sumoylation have been identified in regulation of ion channel activity [41,42] and metabolic pathways [43,44]. Because of this pleiotropic ability to modify numerous proteins and affect a wide range of cellular processes, sumoylation is an attractive target for pathogens to use in modulating the cellular environment to favor pathogen replication and/or maintenance. There are numerous examples of viral proteins that are sumoylated (Table 1), so utilization of this host system by viruses to regulate viral protein function is well documented and will not be further explored in this review (for recent review see [45]). In contrast, recent examples have been described where pathogens alter host sumoylation, either globally or for host specific targets, and the following sections will examine mechanisms by which both viral and bacterial pathogens perturb the host sumoylation process.

**Table 1:** Sumoylated Viral Proteins.

Virus	Protein	Reference
Adenovirus (Ad)	E1B-55K	[127]
Adeno-associated virus (AAV)	Rep 78	[128]
Cytomegalovirus (CMV)	IE1	[108]
	IE2	[116]
Epstein-Barr Virus (EBV)	Zta (BZLF1)	[105]
	Rta (FRLF1)	[132]
	EBNA3C	[133]
Hepatitis Delta virus (HDV)	HDAg	[134]
Human herpesvirus 6 (HHV6)	IE1B	[136,137]
Human immunodeficiency virus (HIV)	p6	[140]
	Integrase	[126]
Human T-cell leukemia virus (HTLV)	Tax	[129]
Influenza	NS1	[145]
	PB1, NP, M1, NS2	[93]
Kaposi's sarcoma-associated herpesvirus (KSHV)	K-bZIP	[76]
	LANA2	[106]
Moloney murine leukemia virus (MMLV)	CA	[130]
Papillomavirus	E1	[135]
	E2	[138]
	L2	[91]
Parainfluenza virus 5	P	[141]
Picornavirus	EV71 3C	[142]
Severe acute respiratory syndrome coronavirus (SARS-CoV)	N	[139]
Vaccina virus	A40R	[143]
	E3	[131]
Varicella-zoster virus (VZV)	ORF29p	[144]

## 3. Sumoylation and Innate Immunity

Immunity to pathogens consists of innate and acquired responses that collectively block or diminish infections and usually leads to resolution of acute disease. Upon first exposure to a new pathogen the innate responses, primarily mediated through induction of interferon and cytokines, are a critical initial defense that dampens pathogen growth until pathogen-specific antibodies and T-cells are developed [46]. The innate response is triggered by recognition of pathogen-specific molecules by the Toll-like receptor (TLR) system which then initiates a signaling cascade that activates IRFs 3, 5, and 7 via phosphorylation; the activated IRFs turn on interferon transcription. Two of the TLRs, RIG-1 [47] and MDA5 [48], and an adaptor protein in the TLR signaling pathway known as Pellino-1 [49] have recently been shown to be sumoylated proteins. Sumoylation of RIG-1 enhanced its association with Cardif and led to increased expression of interferon β. Likewise, MDA5 sumoylation also increased interferon production, indicating that sumoylation is a positive regulator of these two TLR pathways and possibly other TLRs. For both MDA5 and RIG-1, sumoylation and the subsequent increased interferon production repressed viral replication, while reducing overall sumoylation led to increased viral replication. These results suggest that viral down regulation of sumoylation would be beneficial to avoid triggering a strong innate response.

Downstream of the TLRs, sumoylation of both murine IRF3 and IRF7 in response to viral infection has been reported [50]. Interestingly, sumoylation defective mutants of either protein led to increased interferon gene expression after viral infection suggesting that sumoylation is a negative regulator at this stage of the pathway. However, a subsequent study examining endogenous human IRF3 came to the opposite conclusion [51]. Ran *et al*. showed that sumoylation of human IRF3 competed with ubiquitination and protected IRF3 from degradation. When sumoylation of IRF3 was elevated by reduction in the levels of the SENP2 SUMO protease it resulted in decreased viral replication, suggesting that sumoylation was a positive regulator of innate immunity. Whether these conflicting results reflect differences in human *versus* murine IRF3 or differences in the experimental approaches, it is clear that sumoylation is a contributor to the regulatory process that governs the initiation of the interferon response. Consequently, it is likely that viruses have evolved mechanisms to thwart or usurp this upstream portion of the pathway as well as the documented viral effects on downstream effectors such as PKR and 2’,5’-oligoA synthetase [52]. While there are not yet examples of viruses specifically targeting the sumoylation of TLRs or their signaling pathways, several viruses can alter global cellular sumoylation (see sections below) which could impact the functionality of the innate immune response.

In addition to classical innate immunity mediated through interferons and cytokines, the concept of intrinsic immunity has developed in recent years [53]. Unlike the induction cascade required to activate innate immunity, intrinsic immunity operates through pre-existing proteins that act to repress viral infection. Several members of the TRIM family of proteins have been shown to participate in intrinsic immunity, and while some can be up regulated by interferon, their anti-viral activity does not directly require interferon [54]. There is now mounting evidence that sumoylation is important in directing TRIM proteins to their targets. For example, the cytoplasmic TRIM5α protein can block certain retroviral infections [55,56]. A recent publication demonstrated that TRIM5α has three potential SUMO-interacting motifs (SIMs) and that the anti-viral activity of TRIM5α requires SIM1 and SIM2, but not SIM3 [57]. Mutations in the murine leukemia virus (MLV) capsid antigen (CA) that blocked CA sumoylation abrogated the ability of TRIM5α to restrict MLV replication. From these results the authors propose that restriction by TRIM5α, at least in part, involves direct binding of TRIM5α to sumoylated CA through the SIM1 and SIM2 regions in TRIM5α. This SUMO-SIM facilitated interaction presumably then leads to some change in capsid structure that disrupts the normal infection process and restricts viral infection.

Another member of the TRIM family, PML, has general intrinsic anti-viral activity against both DNA and some RNA viruses [58]. It has generally been observed that cells with decreased or absent PML have enhanced viral replication [59,60,61,62] and that PML-/- mice are more susceptible to certain viral infections [63]. Consistent with an anti-viral role for PML, a large number of viruses specifically target PML NBs for disruption and/or degradation, and abrogation of this viral function typically impairs viral reproduction. The interplay between viruses and PML NBs has been discussed extensively in several recent reviews [45,58,64], however, some recent examples that highlight the role of sumoylation in the virus-PML interaction are presented below and in section 4.4.

Elegant studies from Roger Everett’s group found that PML and other NB proteins, such as Sp100 and Daxx, have SIM motifs that are required for recruitment of these proteins to herpes simplex virus (HSV) replication foci [65]. Absence of the SIM motif does not affect PML mobility or normal assembly into NBs, so the SIM motif seems to function primarily to direct the formation of NB protein complexes on viral replication complexes. These NB complexes are transient in wild type HSV infection due to their disruption by the early gene product ICP0. However, an HSV ICP0 null mutant is normally highly defective for replication unless PML is depleted, suggesting that recruitment of the NB proteins creates a repressive environment at the replication foci that would block viral replication unless overcome by ICP0 [65,66]. Reintroduction of wild type PML decreases viral production by the ICP0 mutant while reintroduction of a SIM minus PML does not, strongly implicating the SIM motif in targeting PML to the replication foci [65]. Consistent with the importance of the PML SIM, viral replication foci were found to have a significant deposition of SUMO1 and SUMO2/3 which could serve as the platform for SIM binding. Furthermore, knockdown of the sole SUMO conjugating enzyme, Ubc9, reduces accumulation of SUMO1 and SUMO2/3 signals at the viral foci suggesting that de novo sumoylation is needed for the accretion of the sumoylated proteins at these sites [67]. Ubc9 knockdown also results in greatly enhanced replication of the ICP0 null mutant which is again consistent with sumoylation contributing to intrinsic anti-viral activity. Based on these observations the authors proposed that sumoylated proteins assemble on the viral genomes and serve as the signal to recruit PML and other SIM containing NB proteins to viral replication complexes. Assembly of PML, Sp100, and Daxx would normally inhibit HSV replication if not counteracted by the ICP0 protein (see below). Presumably this SIM-SUMO dependent recruitment could be a general mechanism that accounts for PML anti-viral activity on various other invading viral genomes. What the actual sumoylated proteins are and how they might be directed to the nascent viral replication foci is unknown, but could be related to the viral genomes eliciting the cellular DNA damage response.

Not only is the SIM-SUMO interaction important for directing the anti-viral activity of PML NBs, there is increasing evidence that viruses use SIM-SUMO interactions to thwart the effects of PML. PML is itself sumoylated at three sites [68], so PML accumulation at viral replication foci would contribute to the concentration of SUMO groups localized at these foci. Recent studies on herpes varicella-zoster virus (VSV) ORF61 protein showed that ORF61 has a functional SIM motif in the C-terminal domain, and that mutation of this SIM motif significantly impaired the ability of ORF61 to disperse NBs and strongly reduced co-localization of ORF61 with PML NBs [69]. Surprisingly, mutation of this SIM motif did not impair viral replication in permissive cells *in vitro*, but was required for pathogenesis and skin lesions in a SCID mouse model with human skin xenografts. While the mechanism by which ORF61 disperses PML is still unclear, these results strongly suggest that ORF61 targeting to NBs is mediated through the ORF61 SIM interacting with sumoylated proteins, possibly sumoylated PML.

Similar to the ORF61 story, the herpes simplex virus (HSV) ICP0 protein has several putative SIM-like sequences (SLSs), and SLS-4, -5, and -7 contribute to SUMO binding, to degradation of SUMO modified PML isoforms, and to the biological ability of ICP0 to overcome intrinsic anti-viral defenses [67]. Early in infection, unknown SUMO conjugates, along with PML, are recruited to viral genomes that can be visualized as punctate foci in the nucleus. ICP0 is subsequently recruited to these foci in a SUMO2/3-dependent fashion, suggesting that recruitment depends on SIM-SUMO interactions. This ICP0 recruitment does not require PML, so presumably the unknown sumoylated proteins that accumulate with the viral genomes are sufficient to direct ICP0. As for the ORF61 protein, it appears that SUMO moieties are critical signals that mediate localization of ICP0 to the viral genomes in order to counteract the repressive effect of PML and NB proteins. While a number of other viral proteins also disrupt NBs, the role of SIM motifs for targeting these proteins has not been reported; further discussion of NB disruption can be found in section 4.4.

In summary, there is growing evidence that many host proteins involved in innate and intrinsic immunity are regulated by sumoylation, thus their function could be dysregulated by viral attacks on the sumoylation system. Alternatively, the SUMO moieties on these host proteins can serve as targeting signals to recruit viral proteins containing one or more SIM sequences. These SUMO-SIM interactions are critical for directing some viral proteins to cellular defense complexes, such as PML NBs, where the viral proteins can subsequently counteract the defense mechanism. Consequently, sumoylation appears to be a highly important component of viral strategies for overcoming host defenses early in infection.

## 4. Mechanisms of Pathogen Impact on Host Sumoylation

**Figure 1:**
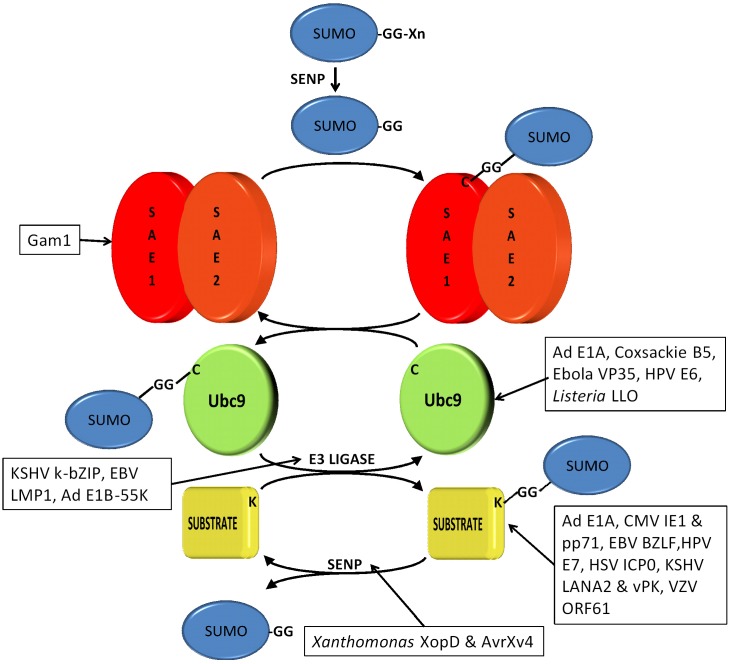
Pathogens and the sumoylation pathway. The enzymology of the sumoylation is shown from the processing of the SUMO precursor to the final conjugation/deconjugation of substrates by the SUMO protease, SENP. Pathogen proteins that act at various steps in the sumoylation pathway are shown in boxes and are discussed in the text.

The previous section detailed some of the recent work defining the interplay of viruses and sumoylation in the innate and intrinsic anti-viral response. However, given the pleiotropic roles for sumoylation in various cellular processes, it is likely the pathogens modulate sumoylation to alter many other facets of the cellular environment, not just immune response. Theoretically, pathogens could mimic or co-opt any step in the sumoylation process in order to alter the sumoylation status of viral or host proteins, and there are now excellent examples for many steps in the pathway (Figure 1). The following sections will describe examples where viral proteins perturb host sumoylation, either globally or for specific targets.

### 4.1. Pathogen Proteins that Mimic Sumoylation Enzymes

There are now well documented examples of pathogens that express proteins that mimic either SUMO proteases or SUMO ligases. *Xanthomonas campestris*, a plant pathogen, uses a type III secretion system to inject effector proteins into host cells during infection. Among the injected effectors are two proteins, XopD and AvrXv4, that both decrease overall host sumoylation [70,71,72]. XopD resembles the yeast SUMO protease, Ulp1, can deconjugate SUMO from substrates *in vitro* and *in vivo*, and causes an overall decrease in host sumoylation when exogenously expressed [70]. In a subsequent study it was confirmed that XopD is a virulence factor in tomatoes and that virulence at least partially requires the protease activity, though the critical target(s) for desumoylation were not identified [72]. A second *Xanthomonas* protein, AvrXv4, resembles a cysteine protease and causes desumoylation in plants [71]. However, *in vitro* SUMO protease activity by AvrXv4 could not be demonstrated raising the possibility that AvrXv4 is not directly a SUMO protease but somehow influences cellular SUMO proteases. Like XopD, AvrXv4 has a role in virulence, and the combined results strongly suggest that *Xanthomonas* is using SUMO deconjugation to alter the host cell environment to favor bacterial infection.

There have been reports of two viral proteins that exhibit SUMO ligase activity, the Kaposi’s sarcoma-associated herpes virus (KSHV) K-bZIP protein [73] and the adenovirus E1B-55K protein [74,75]. K-bZIP is a nuclear transcription factor that is a strong repressor when sumoylated at lysine 158 [76]. Phosphorylation at threonine 111 prevents sumoylation and converts K-bZIP to a strong transcriptional activator [77]. Chang *et al*. showed that K-bZIP has a SIM motif at residues 73–77 that binds SUMO2/3 but not SUMO1 [73]. They also showed that K-bZIP acts as a SUMO2/3-specific SUMO ligase that enhances sumoylation of its binding partners, including p53 and pRB, and that the ligase activity required the intact SIM motif. Additionally, expression of K-bZIP in an inducible cell line resulted in a global increase in SUMO2/3 conjugates with no effect on SUMO1 conjugates, illustrating that K-zBIP could have wide-spread effects on cellular events through changing the sumoylation status of numerous proteins. Exactly how this benefits viral reproduction is not yet determined, but clearly K-bZIP is both regulated by sumoylation and is able in turn to regulate sumoylation of host proteins.

In contrast to K-bZIP, the adenovirus E1B-55K protein appears to be a SUMO1-specific SUMO ligase [74]. It was originally reported that E1B-55K enhanced p53 sumoylation *in vivo* but lacked SUMO ligase activity *in vitro*, suggesting that it might be recruiting a cellular ligase to the E1B-55K/p53 complex [75]. However, a subsequent study showed both *in vivo* and *in vitro* enhancement of p53 sumoylation confirming that E1B-55K is itself SUMO ligase [74]. This ligase activity may be specific to p53 as E1B-55K did not increase sumoylation of pRB [75]. Interestingly, E1B-55K is itself sumoylated at lysine 104, and mutation of this residue reduces ligase activity on p53. Pennella *et al*. found that this same SUMO conjugation site mutation also impaired E1B-55K association with PML NBs and the E1B-55K-dependent export of p53 to cytoplasmic aggresomes [74]. Additionally, they demonstrated that sumoylation of p53 reduced its intracellular mobility and increased its tethering to NBs resulting in enhanced nuclear export of p53. Based on these observations they proposed that the SUMO ligase activity of E1B-55K is a mechanism that contributes to repression of p53 function by increasing p53 sumoylation which facilitates the association of p53 with NBs and its subsequent export from the nucleus.

### 4.2. Pathogen Proteins that Act as STUbLs

STUbLs (**S**UMO **T**argeted **Ub**iquitin **L**igases) are a group of ubiquitin ligases that contain SIM motifs that target the ligases to sumoylated proteins [78]. A recent report by Boutell *et al*. demonstrated that the herpes simples ICP0 protein has properties suggestive of a STUbL [67]. ICP0 has 7 predicted SIM-like sequences (SLSs), and at least some of them are functional for SUMO binding. In yeast two-hybrid assays ICP0 interacts in a SLS-4 dependent manner with SUMO2/3 but not SUMO1. In contrast, in *in vitro* pull-down assays the C-terminal portion of ICP0 binds SUMO1 and not SUMO2/3; SLS-5 appeared to be important for this SUMO1 interaction, but other sequences may also contribute. The discrepancy between the two-hybrid and pull-down assay results was not resolved, but together they support the interpretation that ICP0 may make interactions with all the SUMO isoform. In addition to SUMO binding, ICP0 can specifically ubiquitinate poly-SUMO chains *in vitro*. This activity requires both the RING domain and an intact SLS-4 sequence. ICP0 SLS-4 mutants retain full ubiquitinating activity on other substrates, so the SIM motif appears necessary only for directing ICP0 to sumoylated substrates. Consistent with the *in vitro* results, expression of ICP0 in a stable cell line resulted in a decrease in global SUMO conjugates, including sumoylated forms of PML, and this activity was reduced though not entirely eliminated in an SLS-4 mutant. These results suggest that ICP0 is ubiquitinating sumoylated proteins and targeting them for proteasomal degradation. Thus, it appears that the SLSs of ICP0 can redirect the intrinsic ubiquitin ligase activity to sumoylated proteins, such as PML, which may contribute to ICP0’s ability to overcome intrinsic cellular resistance to HSV. Given the growing number of known viral ubiquitin ligases [79], it would be surprising if others were not also STUbLs.

### 4.3. Pathogen Proteins that Target Sumoylation Enzymes

A large number of pathogen proteins have been shown to target the sumoylation system in order to modulate overall sumoylation levels in the host cell. Many pathogen express proteins that target Ubc9, the SUMO conjugation enzyme, but in some cases other sumoylation enzymes are also targeted. The one known example of a pathogen protein that targets the SUMO activating enzyme (SAE) is the Chicken Embryo Lethal Orphan (CELO) adenovirus GAM1 protein [80]. GAM1 is critical for CELO replication [81], and it was quickly shown that GAM1 expression decreased sumoylation of the HDAC1 histone deacetylase [82]. A subsequent study revealed that GAM1 expression leads to a dramatic decrease in total SUMO conjugated products in the cell, that GAM1 binds the SAE1/2 SUMO activation enzyme and inhibits its activity *in vitro*, and also strongly reduced intracellular levels of SAE1, SAE2, and Ubc9 [80]. Boggio *et al*. further demonstrated that GAM1 binds the cullin proteins, Cul2 and Cul5, and recruits the Cul2/5-EloB/C-Roc1 ubiquitin ligase complexes to SAE1/2 [83]. *Invitro* the recruitment of the ubiquitin ligase causes enhanced ubiquitination of SAE1and *in* *vivo* a GAM1 mutant that cannot bind the cullins is unable to decrease SAE1/2 levels, implicating ubiquitin mediated proteasomal degradation as the mechanism by which the SUMO activating enzyme is reduced in the presence of GAM1. However, SAE2 is not directly ubiquitinated in the ligase complex, so the loss of SAE2 appears to be due to its destabilization when not in complex with SAE1. The net result of GAM1 action on SAE1 is dramatic reduction in levels of both SAE1 and SAE2 with the resultant cessation of *de novo* sumoylation. Sumoylation is generally associated with transcriptional repression [84], so global loss of sumoylation should result in a much more transcriptionally robust cellular environment for the virus [85].

There are two known examples of viral proteins targeting SUMO ligases, the human papillomavirus (HPV) E6 protein [86] and the Ebola virus VP35 protein [87]. The HPV E6 binds the PIASy SUMO ligase to inhibit sumoylation of PIASy substrates [86]. E6 did not induce degradation of PIASy so the inhibition of activity appears to be a direct result of binding. This binding and inhibitory activity was restricted to the high risk HPV 16 E6 protein and was absent in the low risk HPV11 E6 protein. Interestingly, the ability of E6 to overcome PIASy induced senescence was not dependent upon p53 so must be operating through other PIASy targets, such as pRB. The ability of the 16E6 protein to inhibit sumoylation-promoted induction of senescence through the pRB pathway would clearly be of value for viral infection and possibly transformation.

In contrast to the HPV 16E6 protein, the Ebola VP35 protein stimulates the ligase activity of its target, PIAS1. PIAS1 is an endogenous SUMO ligase for IRF7. Sumoylation of IRF7 decreases its transcriptional activity on the interferon β promoter and may be part of a normal feedback process to attenuate the interferon response and limit inflammation. In co-immunoprecipitations VP35 was found complexed to both PIAS1 and IRF7, and VP35 expression led to enhanced sumoylation of IRF7 that reduced its transcriptional activity. When co-expressed with a PIAS1 inactive mutant VP35 was unable to enhance sumoylation of IRF7, indicating that the VP35 effect on IRF7 is mediated through PIAS1 alone. Normally IRF7 is sumoylated at lysine 406, but VP35 induced sumoylation at multiple lysines, even in a lysine 406 mutant, suggesting that VP35 is causing promiscuous sumoylation of this target to ensure its transcriptional repression. Similar effects were seen with IRF3. Consequently, it appears that Ebola is using VP35 to down regulate the innate immune response by hypersumoylating two transcription factors involved in interferon induction to reduce their transactivation capacity and decrease interferon production.

While there are only a limited number of viral proteins known to target either SAE or SUMO ligases, pathogen proteins affecting Ubc9 are more numerous, suggesting that Ubc9 is a very effective cellular target for manipulating sumoylation either globally or for specific substrates. Note that most sumoylated proteins, including pathogen proteins, interact with Ubc9 as part of the sumoylation process, however, the focus in this section will only be on interactions that alter or potentially alter Ubc9 function. Examples have now been documented for both global sumoylation increases and decreases mediated by different pathogens, so both scenarios apparently can provide benefit in a pathogen-specific manner. The two examples of pathogen-dependent decreases in sumoylation via effects on Ubc9 are the *Listeriamonocytogenes* LLO protein [88] and the HPV E6 protein [89]. *L. monocytogenes* is a facultative intracellular human pathogen associated with foodborne illnesses. Infection of HeLa cells with *L. monocytogenes* results in a general decrease in overall SUMO1 and SUMO2/3 conjugates. This decrease does not require bacterial entry into the cells, but is dependent on a known virulence factor, the listeriolysin O (LLO). Administration of purified LLO to cells also decreased sumoylation, but had no effect on *invitro* sumoylation indicating that LLO was not directly inhibiting enzyme activity. Cell culture studies revealed that LLO was reducing intracellular Ubc9 levels with no effect on SAE, and a similar LLO-dependent decrease in Ubc9 was observed in an infected mouse model. Over expression of SUMO1 or SUMO2 in infected HeLa cells led to increased overall sumoylation and significantly decreased numbers of intracellular bacteria at seven hours post-infection, indicating that sumoylation is detrimental to bacterial reproduction and must be reduced to allow optimal pathogen growth. Mechanistically, the reduction of Ubc9 was not at the transcriptional level and could not be prevented by proteasome inhibition. However, an aspartyl protease inhibitor partially restored Ubc9 levels in the presence of LLO, suggesting that LLO may be acting through a cellular protease that can target Ubc9. Related toxins from *Clostridium perfringens* and *Streptococcus pneumonia* also reduced Ubc9 levels, indicating that host sumoylation may be generally restrictive to other bacterial pathogens and that these pathogens have evolved mechanisms to counteract this host system.

The high risk HPV E6 proteins, 16E6 and 18E6, also reduce both intracellular Ubc9 levels and overall sumoylation; low risk 11E6 had no effect on Ubc9 or sumoylation, but other low risk E6 proteins were not tested so the generality of this observation is not known [89]. High risk E6 proteins are known to induce proteasomal degradation of several host proteins via direct binding of the target and recruitment of a host ubiquitin ligase called E6AP [90]. Consistent with a possible proteasomal degradation mechanism, both 16E6 and 18E6 bound Ubc9 in pull-down assays. Additionally, the *in vivo* reduction in Ubc9 levels was E6AP dependent, and there was no effect at the transcript level. However, standard proteasomal inhibitors were relatively ineffective in restoring Ubc9 levels so the mechanism of degradation remains uncertain. Furthermore, the critical sumoylated substrates have not been identified and the direct advantage to the virus of reduced sumoylation has not been established, so the biological contribution of this E6 activity is uncertain. Nonetheless, the fact that E6 targets two sumoylation enzymes, Ubc9 and PIASy (discussed above), suggests that modulation of sumoylation is important to the viral life cycle. Interestingly, another HPV protein, the minor capsid protein L2, increases global host sumoylation, though the mechanistic basis for this increase has not been explored and may not involve Ubc9 [91]. Since L2 is a late protein and E6 an early protein, it is possible that both up and down regulation of sumoylation are required at different stages of the viral cycle.

As opposed to the global reduction in sumoylation by LLO and E6, the Epstein-Barr virus (EBV) LMP1 oncoprotein targets Ubc9 and causes enhanced global sumoylation [92]. LMP1 is the latent membrane protein and possesses six transmembrane domains and a 200-amino acid C-terminal cytoplasmic tail containing three CTAR motifs. Bentz *et al*. showed that LMP1 binds Ubc9 through CTAR3 and that the interaction requires enzymatically active Ubc9. The LMP1-Ubc9 interaction leads to an increase in total SUMO conjugates with SUMO1, SUMO2, or SUMO3, and this increase is eliminated by a CTAR3 deletion. Abrogating the increase in sumoylation with the CTAR3 deletion does not cause an effect on host cell growth, but does reduce the ability of LMP1 to stimulate cell migration in a scratch assay, indicating that LMP1’s ability to increase sumoylation does have biological implications. Mechanistically, LMP1 did not cause any change in Ubc9 protein levels so the authors proposed that LMP1 may be acting as a SUMO ligase, though the critical targets for the migration effect remain to be determined. A similar global increase in sumoylation was also observed during influenza A virus infection of HEK293, A549, MDCK, and Vero cells [93]. The viral protein mediator was not identified, and there was no change in intracellular levels of Ubc9, SAE1, or SAE2, so the viral target that causes this increase in sumoylation is unknown. However, the increase in sumoylation could not be mimicked by interferon treatment alone and did not occur with UV inactivated virus, suggesting that one or more viral products produced during active infection were responsible. It will be interesting to see if this effect is somehow mediated through Ubc9 as for LMP1 or if it involves a different mechanism.

An intriguing but more limited viral effect on Ubc9 has recently been reported for the adenovirus E1A oncoprotein [94]. Interaction of E1A to Ubc9 was first reported in 1996 [25], and Yousef *et al*. extended those studies by exploring the interaction in detail [94]. The authors found that the Ubc9 interaction motif on E1A is the sequence EVIDLT in the conserved region 2 (CR2) domain. While this sequence resembles a SIM motif, sumoylation of Ubc9 was not required for the interaction with E1A. Furthermore, unlike the previous examples discussed, E1A interaction with Ubc9 did not appear to affect overall sumoylation *in vivo* nor did it affect *in vitro* sumoylation of two test substrates, HDAC4 or E2-25K proteins. Analysis of Ubc9 mutants indicated that E1A binds to Ubc9 in the N-terminal region involving sequences that overlap but are not identical to those involved with SUMO binding by Ubc9. Since SUMO binding by Ubc9 is critical for formation of SUMO chains (poly-sumoylation), E1A was tested for an effect on poly-sumoylation in a yeast assay. Ulp2 deleted yeast cannot grow at 37 °C due to toxic accumulation of poly-sumoylated proteins, but wild type E1A is able to restore efficient growth while a Ubc9 non-binding E1A mutant cannot. The authors concluded from this result that while E1A does not appear to grossly alter the mono-sumoylation pattern that it can inhibit poly-sumoylation which may affect the function of specific host proteins. They also speculated that E1A binding might affect the ability of Ubc9 to be sumoylated at lysine 14. Sumoylation at this residue alters Ubc9 substrate specificity [95], so this could also have effects on sumoylation of host proteins. How these subtle E1A effects on Ubc9 contribute to viral fitness will require further study.

Lastly, while a specific viral protein mediator has not yet been established, it is worth mentioning another example by which host sumoylation could be perturbed. Coxsackievirus B5 (CVB5) infection of HeLa cells does not alter Ubc9 levels, but causes a dramatic dispersal of Ubc9 from perinuclear aggregates into a diffuse signal throughout the cell [96]. How re-localization affects SUMO conjugates, either globally or for specific substrates, was not examined, but it seem likely that this dispersal of Ubc9 is another viral mechanism for altering sumoylation of host proteins. In summary, it is clear from the examples discussed that both viral and bacterial pathogens have evolved diverse mechanisms to target Ubc9 and influence the host SUMOeome.

### 4.4. Pathogen Proteins that Modulate Sumoylation of Specific Host SUMO Substrates

The previous section described pathogens and pathogen proteins that targeted sumoylation enzymes to cause broad effects on multiple host SUMO substrates. This section will focus on how pathogens modulate sumoylation of specific target proteins. As might be anticipated, the target proteins identified to date include critical host cell regulators of growth or pathogen resistance, such as p53, pRB, PML, and Daxx. All of these host proteins are known to be regulated by sumoylation, so it not surprising that pathogens have developed strategies to dysregulate these proteins through modulation of their sumoylation status.

One of the important host cell proteins whose sumoylation is specifically impacted by pathogen proteins is the cell cycle regulator, pRB [97]. pRB is a tumor suppressor that inhibits cell cycle progression by blocking E2F activity until phosphorylation of pRB by cyclin kinases at late G1/early S phase releases E2F [98]. Ledl *et al*. showed that pRB is sumoylated at lysine 720 in the B-box motif of the so called pocket region, and that the hypophosphorylated form of pRB is preferentially sumoylated [97]. Lysine 720 is part of a “lysine cluster” that contributes to interaction with LxCxE containing proteins such as the adenovirus E1A protein and the papillomavirus E7 protein. Both E1A and E7 bind pRB and displace E2F to stimulate cellular entry into S phase which is critical for viral replication. As suspected from the known binding surfaces, addition of E1A protein to an *in vitro* sumoylation reaction prevents pRB sumoylation, and this inhibition requires direct E1A-pRB interaction. Similarly, i*n vivo* expression of either E1A or E7 inhibits pRB sumoylation. Lastly, they showed that sumoylation of pRB decreases its repressive activity on an E2F driven promoter, indicating that sumoylation is an actual regulator of pRB and that E1A or E7 inhibition of sumoylation would have a functional effect. Whether or not this effect on pRB function is an irrelevant consequence of E1A or E7 protein binding or actually contributes to creation of the permissive environment has not been investigated.

p53 is another cellular tumor suppressor that can elicit both cell cycle arrest and apoptosis in response to infection, so it is a primary target for many viral proteins that seek to limit p53’s functions [2]. As discussed in section 4.1, the adenovirus E1B-55K protein is a SUMO ligase that specifically targets p53 and enhances its sumoylation with SUMO1 [74]. This viral enhanced sumoylation contributes to p53 localization and tethering in PML NBs which results in p53 export and accumulation in cytoplasmic aggregates where it is nonfunctional. This clearly constitutes viral hijacking of the sumoylation system to suppress the activity of a host defense protein.

Another interesting and so far unique example of a viral protein influencing the sumoylation of a specific host protein is that of the Kaposi’s sarcoma-associated herpesvirus (KSHV) viral protein kinase (vPK/ORF36) and the host KAP-1 protein [99]. KAP-1 is a transcriptional repressor that recruits chromatin remodeling proteins, such as HDACs, to inactivate cellular promoters [100]. KAP-1 is sumoylated at three lysines, and the conjugated SUMO moieties contribute to binding of the chromatin remodeling proteins and hence the repressive function of KAP-1 [101]. KAP-1 is also phosphorylated, and phosphorylation of serine 824 reduces sumoylation at all three lysines and thus decreases the repressive activity of KAP-1 [102]. Chang *et al*. subsequently presented evidence that KAP-1 is involved in the switch between latent and lytic growth for KSHV [99]. Importantly, they showed that the viral kinase could phosphorylate KAP-1 leading to decreased KAP-1 sumoylation and decreased occupancy of KAP-1 on viral promoters. These results point to a model where KSHV uses phosphorylation of KAP-1 by the vPK as a mechanism to antagonize KAP-1 sumoylation and overcome KAP-1 repression, thus favoring lytic replication over latency.

Finally, many viruses have developed mechanisms to disrupt sumoylation of PML and associated proteins such as Daxx. Given the important role of PML NBs in innate immunity it is to be expected that viruses would need to overcome this defense in order to establish a productive infection. The finding, that PML assembly into NBs and recruitment of associated proteins is highly dependent upon PML sumoylation [103,104], offers a rationale for why many viruses attack the sumoylation status of PML. There is currently a large list of viral proteins that are known to disrupt NBs, and this is a particularly prominent feature of the herpesvirus family, including the BZLF protein of Epstein-Barr virus [105], the LANA2 protein of Kaposi’s sarcoma-associated herpes virus [106], human cytomegalovirus IE1 protein [107], the herpes simplex ICP0 [108], and the varicella zoster virus ORF61 protein [69]. Somewhat surprisingly the different herpes viruses utilize different mechanisms to disrupt PML sumoylation. For example, the EBV BZLF protein (also known as Zebra, Z, or Zta) is efficiently sumoylated at lysine 12 and appears to prevent PML sumoylation by out competing PML for a limited supply of free SUMO [105]. Consistent with this model, a lysine 12 mutant of BZLF is impaired in dispersing PML bodies when BZLF is limited compared to PML [109]. In contrast, as discussed in section 4.2, the herpes simplex ICP0 protein appears to be a SUMO-targeted ubiquitin ligase [67]. ICP0 contains SIM motifs that direct it to PML where the ICP0 ubiquitin ligase activity conjugates ubiquitin to PML and enhances its proteasomal degradation. Similar to ICP0, the varicella zoster virus ORF61 protein also possesses SIM motifs that are critical for interaction and dispersal of PML NBs [69]. However, the ORF61 protein alone is not sufficient to degrade PML, suggesting that its known ubiquitin ligase activity is not directly involved in modifying PML, so the precise mechanism for PML dispersal remains uncertain. Like ICP0 and ORF61, the LANA2 protein of KSHV has a SIM motif (residues 474–477) that is critical for interaction with and destruction of PML NBs in B cells [110]. Consistent with a SUMO-SIM interaction, the association of LANA2 and PML not only requires the LANA2 SIM motif, but also lysine 160 of PML which is a known sumoylation site. In addition to disruption of NBs, LANA2 causes PML levels to decrease via proteasomal degradation. These effects are associated with a LANA2 mediated increase in PML modification by SUMO2 with a concomitant increase in ubiquitination of PML. SUMO2/3 modification of PML is known to promote degradation of PML due to ubiquitination by the cellular RNF4 ubiquitin ligase [111], suggesting that this may be the pathway triggered by LANA2 expression. A subsequent study found that LANA2 is sumoylated preferentially by SUMO2 and that a sumoylation deficient mutant of LANA2 was impaired for NB disruption [106]. This observation raises the possibility that SUMO moieties on LANA2 may be interacting with SIM motifs on PML or other proteins to further facilitate the formation of complexes which lead to PML degradation. Lastly, the human cytomegalovirus (HCMV) has two early proteins, IE1 and IE2, that are both localized to PML NBs [107,108]. IE1 induces disruption of NBs without degradation of PML [112], but with the loss of sumoylated forms of PML [113,114]. However, IE1 has no SUMO protease activity *in vitro*, and purified IE1 does not inhibit sumoylation of PML *in vitro* [115]. A model proposed by the authors is that direct interaction of IE1 with PML induces disaggregation of NBs that subsequently exposes released PML to cellular SUMO proteases. In this scenario the desumoylation of PML would be indirectly mediated by this viral early protein. The situation with IE2 is even less clear. The initial observation was that IE2 expressed alone co-localized with NBs though this did not lead to NB disruption [107]. While IE2 is sumoylated [116], interacts with both Ubc9 [117] and PIAS1 [118], and has a SIM motif [119,120], a study by Sourvinos *et al*. found that targeting of IE2 to NBs was via association with viral genomes [121]. Consequently, while IE2 is important for viral replication there is little evidence that it plays a role in overcoming the antiviral effect of PML, and this role may be primarily the purview of IE1. In summary, members of the herpesvirus family express early proteins that target PML NBs, and most of these proteins utilize SIM motifs to facilitate their interaction with sumoylated PML. Many of these PML binding herpesvirus proteins subsequently affect the sumoylation status of PML to promote dissemble of the NBs, though they do so through diverse mechanism.

PML NBs include a variety of other cellular proteins, many of which are also sumoylated such as Sp100 [122] and DAXX [123]. An early report indicated that HSV ICP0 reduced the sumoylation of Sp100 as well as PML [108], and a subsequent study found that ICP0 also induced proteasomal degradation of Sp100 [124]. While the mechanistic basis for the reduction in Sp100 sumoylation has not been explored, it is tempting to speculate that it could be through the same STUbL activity that promotes PML degradation. Likewise, early proteins from other herpesvirus family members that can modulate the sumoylation PML may have similar effects on other sumoylated proteins in the NBs. In addition to early proteins, there is now one well documented study indicating that a cytomegalovirus tegument protein, pp71, enhances sumoylation of Daxx [125]. Sumoylation of Daxx requires direct binding of pp71 but does not require Daxx to be in PML NBs. Furthermore, pp71 does not lead to a general increase in cellular sumoylation so this effect appears restricted to Daxx or a very limited number of substrates. Pp71 did not interact with Ubc9 so it is unclear if pp71 is acting as a SUMO ligase for Daxx or stimulating Daxx sumoylation through some less direct mechanism. Functionally, pp71 could still induce degradation of a Daxx SUMO site mutant so sumoylation is not required for that activity. Additionally, no effect of sumoylation on Daxx transcriptional repression was observed so the biological significance of this pp71 mediated enhancement in sumoylation remains obscure.

Overall, the conclusion from this section is that viruses have evolved many strategies to dysregulate host cell proteins through modulation of their post-translational modification with the SUMO paralogs. This has been most thoroughly explored for PML and related NB proteins that have anti-viral activity, but clearly occurs for other important cellular regulatory proteins such as p53 and pRB. Given the growing appreciation of how widely sumoylation impacts many cellular processes, it is highly likely that other important viral attacks on host protein sumoylation will be identified in the future.

## 5. Conclusions

From modest beginnings as a possibly minor modification system, sumoylation has emerged as a major regulatory network that involves hundreds to thousands of proteins through direct SUMO conjugation and SIM motif-mediated interactions with sumoylated proteins. Sumoylation is now known to be involved in major cellular events including transcriptional regulation, cell cycle control, DNA repair, RNA processing, chromatin remodeling, and nucleocytoplasmic trafficking. Viruses and certain bacterial pathogens must utilize many of these cellular processes to facilitate their own gene expression and genome replication. Consequently, it is not surprising that many pathogens have evolved proteins that are regulated by sumoylation or can usurp certain aspects of the host sumoylation system to reprogram the cellular environment to be more permissive for pathogen persistence or reproduction. The recent finding that sumoylation is involved in regulating aspects of intrinsic and innate immunity, provides another rationale for why pathogens, particularly viruses, target the sumoylation system. The ability to avoid or reduce initial host defenses is obviously critical to establishing a productive infection, and dysregulating sumoylation generally or for specific targets appears to be a common mechanism for pathogens to thwart these defenses.

As presented in this review, there are now a multitude of examples of how pathogens impact the sumoylation system. These range from increasing or decreasing sumoylation of single proteins, usually via direct binding of a pathogen protein, to global increases or decreases in sumoylation. Single protein targets typically are key cellular growth regulatory factors or are critical for host immune response. Global changes in sumoylation induced by pathogens are more dramatic, but also more difficult to understand functionally as identifying the critical targets *versus* irrelevant proteins is challenging. Mechanistically, some pathogen proteins act by mimicking sumoylation enzymes whereas others function by binding to and altering the activity of the endogenous host sumoylation enzymes. An emerging theme is that many of these interactions are mediated or enhanced through SIM-SUMO binding, and in some cases each binding partner has one or more SIM motifs and can also be sumoylated. This raises the possibility of combinatorial interactions that may subtly influence complex affinity, stability, or composition and thus have functional consequences. The next few years are likely to reveal additional important nuances about how pathogens utilize sumoylation to their own benefit as well as identifying many exciting new targets.
